# Biomarkers, isolation methods, and therapeutic implications of breast cancer stem cells

**DOI:** 10.1016/j.cpt.2025.01.006

**Published:** 2025-01-23

**Authors:** Peik Lin Teoh, Nurshafiqah Saini

**Affiliations:** Biotechnology Research Institute, Universiti Malaysia Sabah, Jalan UMS, Kota Kinabalu, Sabah, 88400, Malaysia

**Keywords:** Cancer stem cell, Surface marker, Side population, Resistance, Cancer therapy

## Abstract

Breast cancer metastasis and relapse remain uncontrollable despite significant advancements in early diagnosis and treatment, resulting in increased mortality. Breast cancer is the most frequently diagnosed cancer in women worldwide and has become the leading cause of cancer-related deaths. Cancer stem cells (CSCs) may play significant roles in tumor initiation, maintenance, invasion, relapse, metastasis, and therapy resistance. Although this small, highly heterogeneous, and restricted population of CSCs has been extensively studied, their cellular and molecular physiology remains unclear. Nonetheless, CSCs have increasingly become an attractive therapeutic target for combating advanced, treatment-resistant cancers. This necessitates the development of effective and reliable methods for their isolation and enrichment. This review provides an overview of the key characteristics of breast cancer stem cells (BCSCs) and illustrates their role in therapeutic resistance. Furthermore, it highlights various mechanisms underlying cancer cell adaptability and therapy-induced resistance across different breast cancer subtypes. The commonly used methods for BCSC isolation and identification are also discussed, as they could facilitate a deeper understanding of tumorigenesis, metastasis, resistance, and relapse, consequently contributing to the development of more effective therapeutic strategies for breast cancer.

## Highlights

• We outline the methods used for isolating and enriching breast cancer stem cells (BCSCs).
• These stem cells are isolated and identified using biomarkers and functional assays.
• Therapies targeting these stem cells are developed based on breast cancer subtypes.
• Mechanisms underlying resistance to therapeutic interventions have been reported.
• We present challenges and prospects in BCSC clinical research.

## Graphical abstract

**Figure:**
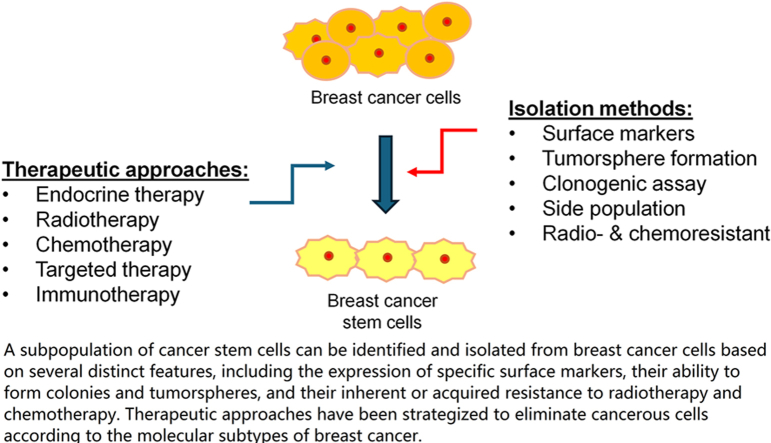

## Introduction

Advancements in scientific research and technology have profoundly expanded our understanding of cancer biology in recent years. These developments have led to the identification of numerous genes, proteins, and signaling pathways involved in cancer initiation and progression. Furthermore, several mechanisms underlying drug resistance have been elucidated, thereby enhancing the development of targeted and effective treatments.^1^ However, the effective treatment of metastatic cancer remains a major challenge despite these scientific breakthroughs and the development of novel, highly precise therapeutic strategies.^2^

Female breast cancer (BC) is the most commonly diagnosed malignant tumor and the leading cause of mortality in women worldwide, with 2.26 million new cases and 684,996 deaths reported.^3^ BC encompasses a group of diseases characterized by mutations in breast tissue cells. These mutations lead to uncontrolled proliferation and the formation of a mass of cancer cells, commonly known as a lump. Additionally, BC exhibits significant heterogeneity, manifesting in diverse histological, molecular, and clinical characteristics. This heterogeneity influences disease progression and results in varying responses to different therapeutic interventions.^4^

Although surgery may be a viable option for certain cancers, such as BC, it poses challenges for metastatic cancer cells. Surgical intervention can create a hypoxic microenvironment, potentially promoting the development of more aggressive relapses.^5^ Additionally, patients undergoing chemotherapy and radiotherapy often experience considerable side effects that further exacerbate their suffering. Furthermore, tumor shrinkage achieved by these treatments is often not sustained over time. In many cases, patients face tumor resistance and recurrence because current therapies fail to eradicate the population of cancer stem cells (CSCs) within the tumor.^6^^,^^7^

CSCs have been recognized as a key factor in therapy resistance in recent years. Their remarkable resilience allows them to survive conventional treatments. This leads to tumor initiation, maintenance, invasion, relapse, metastasis, and resistance to subsequent therapies.^8^ CSCs maintain tumor homeostasis to carefully balance self-renewal and differentiation, thereby sustaining the stem cell population during cancer progression.^9^ Consequently, the biological characteristics of CSCs have been extensively studied, with innovative therapeutic approaches developed to treat advanced, therapy-resistant cancers.^10^ CSC isolation is challenging, owing to their heterogeneity, plasticity, and scarcity, along with the lack of specific biomarkers. Therefore, establishing reliable methods for their identification and isolation is essential for the development of effective treatment strategies.

This review provides an overview of breast cancer stem cells (BCSCs) and the advancements made in their identification and isolation. Given their significant role in treatment resistance and tumor recurrence, the therapeutic implications and prospects of BCSCs are also discussed. This review further highlights the mechanisms and factors related to the adaptability of cancer cells and therapy-induced resistance across different BC subtypes. Additionally, the potential applications of BCSCs in the development of more effective, targeted BC treatments are outlined.

This review compiles findings from various studies. Literature was sourced from PubMed, Scopus, and Google Scholar using keywords like “breast cancer stem cells”, “BCSCs”, “therapy resistance”, “cancer stem cell isolation”, and “breast cancer subtypes” without restrictions on publication year or journal type. The selected articles were analyzed and organized into four themes: (1) characteristics of BCSCs, (2) methods for isolation and identification, (3) potential applications in targeted breast cancer therapies, and (4) challenges and future directions in BCSC research. Although the lack of strict selection criteria may introduce some bias, this approach provides a broad exploration of BCSC-related research. It contributes to a better understanding of tumorigenesis, metastasis, therapy resistance, and the development of more effective, targeted treatments for breast cancer.

## Breast cancer stem cells

Tumors possess small, limited, and rare subpopulations known as BC-initiating cells or BCSCs. These cells share characteristics similar to those of normal stem cells, such as the ability to maintain self-renewal potential across multiple generations and the capacity to differentiate into various cell types.^7^^,^^11^ They can be distinguished by their ability to generate transplantable tumors and restore the heterogeneity of the original tumor.^12^ Despite the effectiveness of chemotherapy and radiotherapy in eliminating most cancer cells, BCSCs may evade destruction due to their resistance to apoptosis and anticancer drugs.^13^ The persistence of tumor-resistant CSCs increases the likelihood of further proliferation and the emergence of additional tumors. This characteristic presents a significant challenge in addressing the relapse and recurrence of BC.^8^

**Figure 1:**
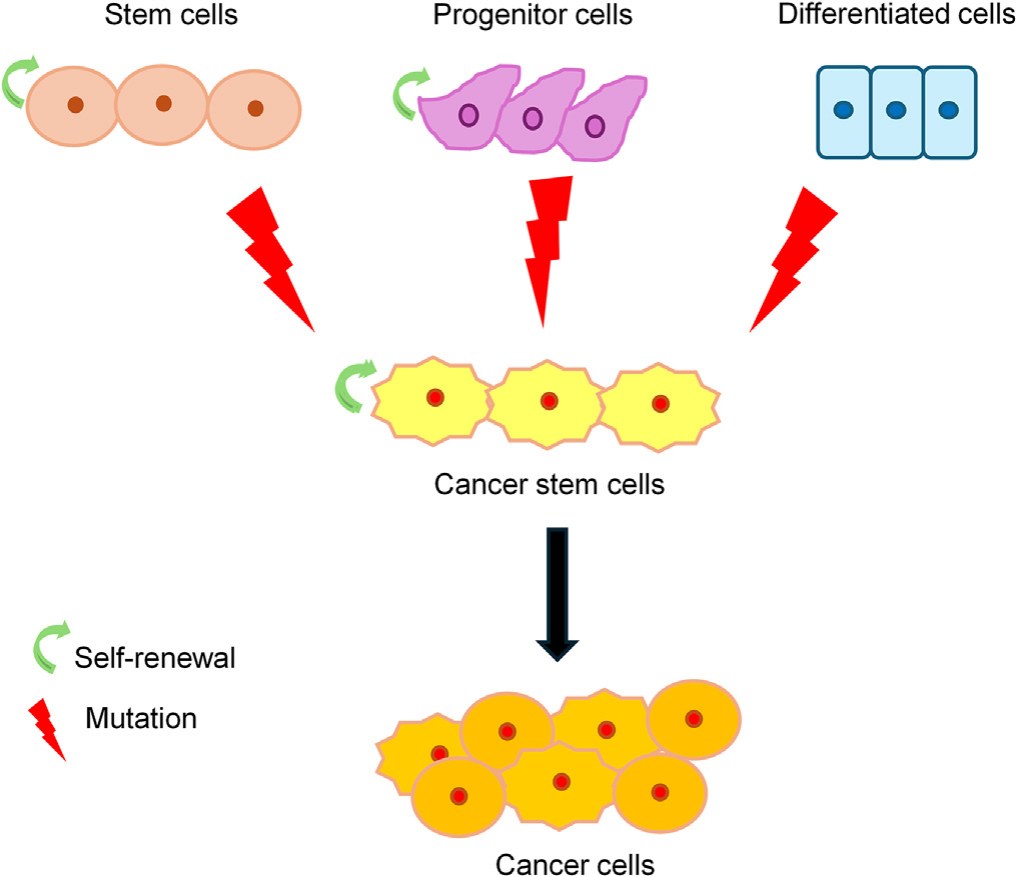
Theories for the formation of cancer stem cells. Cancer stem cells can arise from the mutation of adult stem cells, self-renewal of progenitor cells, or the reversion of differentiated cells to a stem-like state under environmental stress.

Several theories have been proposed to elucidate the origin of BCSCs [Figure 1]. One theory suggests that CSCs may originate from adult stem cells that possess self-renewal characteristics akin to CSCs and may undergo genetic alterations over time, leading to oncogenic transformation. Another theory proposes that CSCs can arise from progenitor cells acquiring self-renewal properties. This hypothesis suggests that multiple mutations in progenitor cells drive cellular transformation and initiate malignancy. Additionally, another hypothesis proposes that CSCs originate from differentiated cells.^14^ Exposure to environmental stressors such as radiotherapy and chemotherapy induces multiple mutations in these differentiated cells, prompting their de-differentiation and re-acquisition of CSC-like properties. This process ultimately leads to the *de novo* generation of BCSCs.^15^ Moreover, changes in the tumor microenvironment can also impact the transformation of non-stem cells into a CSC phenotype. Poli et al.^16^ suggested that myelocytomatosis (MYC)-driven epigenetic reprogramming triggers the conversion of mammary epithelial cells into a CSC-like state.

The theory that CSCs originate from adult stem cells or progenitor cells is the most commonly accepted,^14^ owing to the shared characteristics between CSCs and adult stem cells or partially differentiated progenitor cells. However, these theories continue to provoke debate and controversy among researchers. Nonetheless, this phenomenon can vary among individual tumors. Regardless of the cell of origin, tumors harbor several pools of CSCs that each exhibit unique properties. This diversity reflects their pronounced inherent plasticity.^17^

BCSCs display distinct cellular characteristics that are indicative of their behavior, including self-renewal, differentiation capability, clonogenicity, dormancy, and resistance. They can proliferate and differentiate without attachment to the surface of culture flasks or plates. This attribute is often assessed using the mammosphere formation assay,^18^ where these cells are cultured under low adherence conditions supplemented with epidermal growth factor (EGF) and basic fibroblast growth factor. The resulting spherical cell clusters, termed mammospheres or tumorspheres, serve as a measure of BCSC behavior. They are enriched with various stem cell-associated features, including the activation of stem cell-related signaling pathways.^10^^,^^15^ Spherical cell populations enriched from any tumor tissue are called tumorspheres, whereas those originating from mammary tissue are referred to as mammospheres. BCSCs also often exhibit slow division rates and may display quiescent phenotypes, which can be assessed using label-retention assays. In these assays, cells that retain a high amount of incorporated dye over time indicate limited cell division.^19^

## Isolation and identification of breast cancer stem cells

The identification and isolation of BCSCs within tumor masses can facilitate the elucidation of the molecular mechanisms underlying their origin, self-renewal, differentiation into tumor cells, resistance to radiation therapy and chemotherapy, as well as their potential for invasiveness and metastasis.^20^ However, numerous challenges remain unaddressed. Several approaches have been utilized to identify and isolate CSCs. However, the putative CSCs or CSC-enriched populations obtained through these techniques must undergo rigorous testing via serial xenotransplantation in immunocompromised mice, which serves as the gold standard for CSCs identification.

Flow cytometry and magnetic beads are commonly used to isolate and enrich CSCs. This approach enables simultaneous measurement and analysis of cell characteristics as they pass through a fluid stream illuminated by a light source. This multiparametric method detects the cell surface antigens and metabolic activities of CSCs.^21^ Furthermore, fluorescence-activated cell sorting (FACS) uses fluorescently labeled antibodies to bind to cell surface antigens. It enables the physical sorting of cells into different populations based on fluorescence intensity and type.^22^ This approach, initially used for CSC isolation by targeting surface marker expression such as that of cluster of differentiation (CD)34 and CD38, has successfully identified CSCs in leukemia.^23^ Al-Hajj et al.^24^ subsequently reported the distinct CD44/CD24 expression patterns in tumorigenic BC cells. Later, the sorting of BC cells based on acetaldehyde dehydrogenase 1 (ALDH1) expression has become a widely employed technique, as cells with high ALDH activity possess stem cell-like properties.^25^

Magnetic-activated cell sorting (MACS) offers several advantages compared to FACS, including ease of use, faster processing, reduced cell stress, and cost-effectiveness.^26^ MACS relies on magnetic microbeads conjugated with monoclonal antibodies for cell separation and purification. Cells are incubated with these beads and passed through a column in a magnetic field; the beads bound to cells are retained within the column due to their magnetic properties, whereas unbound cells are washed away. The bound cells can then be recovered through elution after the magnetic field is turned off. MACS is often used to enrich desired cell types or deplete unwanted cells.^27^ It is typically used as a preliminary method for enriching rare cells before they are subjected to FACS. Moreover, MACS has been used to isolate CSCs from complex mixtures with high purity.^28^ For instance, Alhawarat et al.^29^ employed the MagCellect CD24^−^/CD44^+^ Breast Cancer Stem Cell Isolation Kit to sort single CSCs from Michigan Cancer Foundation (MCF)-7-derived mammospheres. Similarly, Farahzadi et al.^30^ utilized MACS to enrich the CD44^+^ CSC population from M.D. Anderson-metastatic breast (MDA-MB)-231 BC cells.

### Breast cancer stem cell biomarkers

BCSCs are typically isolated from heterogeneous populations using flow cytometry or magnetic bead-based methods. These approaches target specific biomarkers expressed on the surface of BCSCs, as outlined in Table 1. In addition, they are widely employed due to their robustness and reliability.^43^

**Table 1:** Biomarkers used for the isolation and identification of breast cancer stem cells.

Biomarkers	Functions	References
CD44	Promotion of invasive and metastatic properties	Yang et al.^31^
CD24	Modulation of drug resistance and phenotypic plasticity in TNBC cells	Deng et al.^32^
CD44^+^CD24^-^	Confers mesenchymal-like BCSCs with quiescent and invasive properties	Liu et al.^33^
ALDH	Increasing the metastatic potential of highly proliferative cells located in the central region of tumors	Liu et al.^33^ and Charafe-Jauffret et al.^34^
CD44^+^CD24^−^ALDH^+^	Possession of the greatest tumorigenic and invasive capabilities	Liu et al.^35^
CD133	Involved in tumor progression and invasiveness	Utnal et al.^36^ and Wright et al.^37^
CD49f	Increases recurrence risk	Ye et al.^38^
CD49f^high^CD61^high^	Enhances tumorsphere formation, tumorigenicity, and drug resistance in Her2/neu-induced mammary tumors	Lo et al.^39^
CD44^+^CD24^−/low^ Lin^−^	Confers tumorigenic potential in immunocompromised mice	Ricardo et al.^40^
CD44^+^CD49f^high^CD133/2^high^	Increases tumorigenic and self-renewal potential in ER^−^ breast cancer	Meyer et al.^41^
CD54 (integrin αvβ3)	Found in aggressive patient tumors with either ER^+^, HER2^+^ or triple negative receptors	Sun et al.^42^

ALDH: Acetaldehyde dehydrogenase; αvβ3: Alpha v beta 3; BCSC: Breast cancer stem cell; CD: Cell of differentiation; CD49f: Cell of differentiation 49f; ER^+^: Estrogen receptor positive; ER^−^: Estrogen receptor negative; HER2^+^: Human epidermal growth factor receptor positive; TNBC: Triple-negative breast cancer.

The CD44^+^CD24^−/low^ expression profile is considered the primary and most reliable marker for distinguishing and isolating tumorigenic BCSCs from non-tumorigenic BC cells. However, CD44 and CD24 markers are often used alone or in combination with other biomarkers because the CD44^+^CD24^−/low^ phenotype may not fully identify all CSC populations within BC cells.^44^ Mesenchymal-like BCSCs characterized by the CD44^+^CD24^−^ phenotype are often predominantly quiescent and localized to the invasive forefront of tumors. In contrast, epithelial-like BCSCs that express ALDH exhibit proliferative behavior and are located in the more central regions of the tumor.^33^ Luminal subtypes are mainly composed of a CD44^−/low^CD24^+^ cell population, whereas basal/mesenchymal BC cell lines predominantly express the CD44^+^CD24^−/low^ phenotype.^40^ Conversely, other basal/epithelial cell lines tend to express both surface markers. ALDH1 activity is commonly observed in human epidermal growth factor receptor 2 (HER2)-overexpressing and basal/epithelial subtypes. These profiles resemble those observed in basal and luminal stem cells within normal breast tissue.^33^^,^^45^ Hence, the combined use of CD44, CD24, and ALDH1 is proposed for identifying BCSCs. Nevertheless, other surface markers such as CD133, CD49f, and CD61 have also been employed, although they are less frequently used.

#### CD44

CD44 is a multifunctional transmembrane glycoprotein that plays a key role in cellular interactions with the extracellular matrix, particularly in binding to hyaluronic acid. Its elevated expression in BCSCs is essential for sustaining their tumorigenic and multipotent properties. It has also been associated with various biological processes, including adhesion, signaling pathways, proliferation, migration, and differentiation. These functions highlight the significance of CD44 in BC progression and its potential contribution to therapy resistance.^31^^,^^46^^,^^47^ For instance, CD44 expression was upregulated by tumor-associated macrophages through the murine double minute (MDM)2/tumor protein (p)53 signaling pathway, consequently contributing to tumor progression and metastasis.^48^

The interactions between CD44 and osteopontin in tumor progression, including invasion, metastasis, tumorigenesis, stemness, angiogenesis, and resistance to chemotherapy and radiotherapy, have also been reported.^49^ For example, CD44 promotes BC metastasis by downregulating nuclear forkhead box A2 (FOXA2) via the protein kinase B (AKT) pathway.^47^ Low CD44 expression allows BCSCs to differentiate into normal cells, whereas its suppression impedes tumor formation. Moreover, it regulates the epithelial–mesenchymal transition (EMT), thereby contributing to tumor initiation and progression.^46^ Furthermore, CD44 expression is significantly higher in triple-negative breast cancer (TNBC) than that in invasive ductal carcinomas.^50^

#### CD24

Another surface marker frequently used for BCSC isolation is CD24. Similar to CD44, it is a glycoprotein. Although CD24 is primarily expressed by immune cells, its expression is often upregulated in human tumors. This glycoprotein regulates cell migration, invasion, and proliferation to facilitate tumor progression and metastasis.^51^ In addition, its overexpression is linked to poor prognosis for luminal A and TNBC subtypes.^52^^,^^53^ For instance, Kwon et al.^52^ demonstrated that increased CD24 expression is associated with lymph node metastasis, consequently decreasing patient survival. Conversely, the loss of CD24 expression in BC cells promotes de-differentiation and the acquisition of hybrid EMT traits, thereby contributing to chemo- and radioresistance.^54^

CD24 modulates chemosensitivity in MCF-7 BC cells. However, its effects vary depending on the drug used. For instance, the suppression of CD24 expression reduces cell sensitivity to 5-fluorouracil but does not alter their sensitivity to *cis*-diamminedichloroplatinum.^55^ Moreover, the low specificity and heterogeneous expression of CD24 may lead to inaccurate isolation of CSCs. The CD44^+^/CD24^−^ phenotype and high levels of ALDH in BCSCs significantly contribute to the recurrence of invasive BC subtypes, owing to their potent capacities for self-renewal and differentiation.^56^ Moreover, Qiao et al.^57^ demonstrated that the transition from a CD44^−^/CD24^−^ phenotype to a CD44^+^/CD24^−^ phenotype holds clinicopathological significance and is linked to metastasis in patients with BC. However, although CD44 and CD24 are clinically relevant markers in tumorigenesis and prognosis markers for patients with TNBC, they are not standalone determinants of prognosis in invasive BC.^58^^,^^59^ Thus, using a combination of biomarkers is generally preferred to improve accuracy and specificity in the identification of target cell populations.

#### Acetaldehyde dehydrogenase 1

ALDH1 belongs to the ALDH enzyme family and is crucial for detoxification. It catalyzes the conversion of acetaldehyde into acetic acid in the cell.^60^ In addition, ALDH1 plays functional roles in cellular proliferation and differentiation. It is particularly involved in stem cell differentiation and is implicated in the metabolism of retinol and retinoic acid. Its expression is often associated with stemness-related markers such as octamer-binding transcription factor 4 (OCT4) and polycomb complex protein (BMI1).^44^

Numerous studies have demonstrated a significant correlation between aldehyde dehydrogenase 1 family member a1 (ALDH1A1) expression and various clinicopathological factors in BC, including tumor size, nodal status, histological grade, and BC subtypes.^61^ Moreover, increased ALDH1 expression is linked to chemoresistance and invasiveness in breast tumors.^62^^,^^63^ Bu et al.^64^ revealed that the stem-like properties of ALDH^+^ cells in TNBC are regulated by the interaction between Kita-Kyushu lung cancer antigen-1 (KK-LC-1) and FAT atypical cadherin 1 (FAT1). This interaction disrupts the Hippo signaling pathway, resulting in the nuclear translocation of yes-associated protein 1 (YAP1) and subsequent activation of *ALDH1A1* transcription, thereby promoting stemness-related characteristics.

#### CD133

CD133, also known as prominin-1, is a transmembrane glycoprotein widely expressed in various human malignancies.^65^ Its expression is associated with invasive ductal BC. Moreover, it is often co-expressed with other putative CSC markers.^36^^,^^66^

The lack of overlap between CD133^+^ and CD44^+^/CD24^−^ subpopulations suggests heterogeneity within BCSCs. A CD133^+^ subset isolated from breast cancer (BRCA)1-deficient mouse mammary tumors exhibited notable abilities in tumorsphere formation, tumor initiation, and drug resistance.^37^ Furthermore, ALDH^high^CD44^+^CD133^+^ cells isolated from TNBC cell lines such as MDA-MB-231 and MDA-MB-468 exhibited elevated proliferation, enhanced colony formation, as well as increased migration and invasion. These characteristics significantly contributed to tumorigenesis and metastasis in murine models.^67^ However, the reduction of ALDH levels effectively diminished the radio- and chemoresistance observed in cell populations harboring this phenotype.^68^

The Thomsen–Friedenreich antigen (CD176) is co-expressed with CD44 or CD133 in a subset of BC-initiating cells, highlighting its potential as a supplementary biomarker for BCSC isolation.^69^ This co-expression suggests its potential for utilization in combination with existing biomarkers such as those outlined in Table 1. This combination may enhance precision in the identification and characterization of these stem-like cell populations.

A consensus on universally recognized CSC-specific markers remains elusive for most cancer types, including BC. Thus, further validation is required to confirm the self-renewal and stemness properties of isolated populations to ensure their accurate identification and characterization. To ensure the efficacy of isolation methods, functional assays are often conducted in parallel to evaluate the ability of cells to form colonies and mammospheres. In addition, their susceptibility to chemotherapeutic agents and radiotherapy can be evaluated. However, tumorigenicity testing in animal models remains the benchmark for confirming the ability of isolated populations to replicate the tumor cell hierarchy and exhibit authentic CSC behavior under physiological conditions. This time-consuming and labor-intensive approach is not feasible in all studies.

### Isolation based on functional assays

In addition to distinctive surface markers, CSCs also possess traits such as self-renewal, quiescence, asymmetric cell division, slow proliferation, elevated ALDH activity, and diminished mitochondrial function. Functional assays that utilize these characteristics for CSC isolation and enrichment offer a straightforward alternative to laborious sample preparation and expensive equipment such as FACS. This method is especially advantageous for limited samples, requiring only a small number of cells for isolation.^70^

#### Spheroid formation assay

Traditional two-dimensional (2D) cell culture models have long been used for screening anticancer drugs *in vitro*. However, these models often fail to replicate the three-dimensional (3D) properties of the tumor microenvironment accurately. Non-adherent or ultra-low attachment 3D culture systems have been developed to address this limitation, allowing cells to proliferate without attachment; this is known as anchorage-independent growth. CSCs typically exhibit rounded morphology, microsize, and an ability to persist as free-floating cells. Tumorspheres grown in these conditions closely mimic the 3D architecture of tumors, consequently providing a more physiologically relevant environment for studying cancer biology and drug responses compared to 2D cell culture methods.^71^

Dontu et al.^18^ demonstrated the efficacy of serum-free nonadherent culture techniques in enriching breast stem cells within primary human mammary epithelial cell populations. These cells formed mammospheres and could differentiate into various mammary epithelial lineages upon introduction into adherent culture. The mammospheres isolated from the MCF-7 BC cell line also displayed protein expression profiles of stem cells and subsequently initiated new tumors when injected into the mammary fat pad of mice.^72^ Grimshaw et al.^73^ later showed the generation of mammosphere colonies from pleural effusions of patients with BC; a markedly high fraction of tumor-initiating cells was observed. Furthermore, stem-like cells obtained from normal breast tissue, malignant primary breast tumors, and MCF-7 cells displayed diverse capacities for mammosphere formation,^74^ highlighting the need for optimizing the suspension culture system used for enrichment.

In addition to serum-free media, various matrices, such as agar-based techniques; natural or synthetic scaffolds such as Matrigel, GrowDex, collagen, and synthetic hydrogels; as well as ultra-low attachment plates, have also been used to establish non-adherent *in vitro* cultures.^75^^,^^76^ Although ultra-low attachment plates are effective, agar-based scaffolds are preferred because of their lower cost.^76^ However, the success rate of isolating CSCs using this technique is modest, typically ranging from 1 to 30%. Moreover, spheroid cells may undergo spontaneous differentiation or apoptosis during serial passaging. Additionally, not all cells generated *in vitro* can effectively develop into tumors upon implantation *in vivo*.^20^^,^^77^

The sphere-forming assay is a valuable standard for isolating and enriching CSCs *in vitro*, owing to its anchorage-independent nature. Morata-Tarifa et al.^78^ isolated CSCs from breast and colon cancer cell lines using a modified protocol termed “differential trypsinization,” which was initially introduced by Owens et al.^79^ This innovative approach is non-aggressive, straightforward, cost-effective, and reproducible compared to the FACS method.

#### Colony forming assay

CSCs can also be isolated through colony forming assays, also known as clonogenic assays.^71^ This quantitative technique measures the ability of a single cell to self-renew and form a colony comprising a tightly packed cluster of ≥50 cells resulting from clonal expansion *in vitro*.^80^ It was initially designed to evaluate the efficacy of radiation or chemotherapeutic agents on cancer cell survival and proliferation. However, this technique has evolved into a versatile tool for probing the stemness of cells isolated from tumors or cancer cell lines.^80^^,^^81^ In addition, colonies originating from CSCs tend to be larger in size and greater in number compared to those derived from non-CSCs. However, the specific characteristics of colonies derived from CSCs can vary depending on factors such as the cancer type, experimental conditions, and the presence of other cell types in the culture.^82^

Using agar to cultivate anchorage-independent cells has become more common as non-adherent 3D culture methods have gained popularity. However, several physical factors can also influence the colony formation assay. For instance, the choice of agar for coating plate wells is critical, as it requires autoclaving and careful cooling to a suitable temperature for pouring. Additionally, maintaining a culture medium with appropriately diluted cells at a temperature conducive to cell viability is crucial to preventing cell death. However, accurately confirming the single-cell origin of each colony can be challenging due to potential errors in cell dilutions, which as often influenced by human factors. Not all cells can thrive in agar medium, as certain types of agar can be toxic, consequently affecting colony formation.^82^ Importantly, the mechanism underlying the formation of clonal spheres from CSCs remains unclear. Therefore, researchers should be cautious of over-interpreting results.^83^

#### Side population assay

As specific surface markers for isolating tumor stem cells are lacking, the isolation of side population (SP) cells has emerged as the primary isolation method.^84^ SP cells are small tumor cell populations within tissues that possess the unique ability to efflux Hoechst 33342 dye. These cells are characterized by their enrichment in stem cell activity, which is marked by elevated clonogenic capacity, tumorigenicity, multipotency, and chemoresistance. Their ability to exclude Hoechst dye is attributed to the elevated expression of adenosine triphosphate (ATP)-binding cassette (ABC) transporters such as ABCB1 and ABCG2. The expression of these transporters is associated with drug resistance in cells that exhibit potential for self-renewal and differentiation.^20^^,^^85^ The active efflux of lipophilic fluorescent dyes prevents the accumulation of SP cells overexpressing ABC transporters, as well as their binding to DNA content. This consequently reduces fluorescence detection. This SP population is typically analyzed through flow cytometry after staining, using dual-wavelength analysis to simultaneously detect the emission signals of Hoechst Red and Hoechst Blue. SP cells are distinguished by their low fluorescence intensity in both channels, which facilitates their separation from the tumor sample.^86^ Therefore, it is useful for isolating and studying CSCs and multidrug-resistant cell populations, which are typically characterized by high ABC transporter activity.

SP cells have been identified in various cancer types, including BC. They exhibit enhanced self-renewal and tumorigenicity upon transplantation into immunocompromised mice. In breast carcinoma, SP cells showed elevated expression of stem cell-related genes such as *ABCG2*, *OCT4*, and *EpCAM* compared to non-SP cells.^87^ However, not all cancers contain SP cells. This indicates that they may represent only a subset of potential CSC populations. Moreover, the SP phenomenon is not exclusive to stem cells; it has also been observed in some differentiated cells within adult tissues.^88^ Stem cells have also been isolated from non-SP cells, suggesting that dye efflux alone may not be sufficient for their detection.^20^ As such, the use of Hoechst 33342 dye for identifying CSCs remains controversial.

### Isolation based on chemotherapy drugs and radiation stimulation

Although chemo- and radiotherapy effectively treat BC, surviving cells often lose their specialized phenotype and transition into a more primitive, stem cell-like state through dedifferentiation. This shift is partly driven by DNA damage repair, reactive oxygen species (ROS), multidrug resistance (MDR), and the activation of stemness pathways, including wingless-related integration site (Wnt)/β-catenin, Notch, and Hedgehog. Treatment-induced stress, inflammation, and hypoxia further enhance the EMT, metabolic reprogramming, and epigenetic changes. As these cells transform from an epithelial to a mesenchymal phenotype, they acquire metastatic capabilities and show increased expression of stemness markers such as Oct4, sex-determining region Y (Sox)2, and CD44.^89^^,^^90^

CSCs possess robust anti-apoptotic mechanisms, protective autophagy, efficient DNA repair systems, and drug transporters. These characteristics enable them to withstand chemotherapy and radiotherapy.^84^ Therefore, enriching CSCs through these treatments is feasible. In addition to the SP assay, resistant cell populations can be distinguished using assays such as the (3-(4,5-dimethylthiazol-2-yl)-2,5-diphenyltetrazolium bromide) (MTT) and determination of 50% inhibitory concentration (IC_50_).^91^ However, achieving BCSC enrichment necessitates varying dosages of chemotherapy drugs and radiation exposure, owing to the differing sensitivities and resistance mechanisms among cancer cell types.^92^^,^^93^

Variation in chemotherapy dosage involves the administration of sub-lethal doses that selectively target and eliminate sensitive non-stem cancer cells while allowing resistant BCSCs to survive and proliferate. In this strategy, the dosage is gradually increased over successive treatment cycles. Alternatively, the approach is tailored to exploit specific drug sensitivities. Thus, this strategy can effectively enrich the stem-like population while minimizing damage to normal tissues. For instance, He et al.^94^ demonstrated that U.S. Food and Drug Administration (FDA)-approved chemotherapy drugs administered to various TNBC cell lines enriched BCSCs by suppressing glutathione S-transferase Mu activity. Similarly, varying radiation exposures, such as fractionated doses or tailored high/low levels, could also exploit the DNA repair mechanisms and intrinsic radiosensitivity of BCSCs. Contrastingly, Inalegwu et al.^95^ reported that fractionated irradiation of MCF-7 BC cells altered the gene regulatory network, leading to a stemness phenotype and increased resistance. Combination therapies involving chemotherapy and radiotherapy, or their sequential administration, have shown potential to synergistically enrich BCSCs. However, experimental validation *in vitro* or *in vivo* is required to determine the optimal dosages and exposure conditions for this approach.

Resistance to chemotherapeutic drugs, endocrine therapies, and HER2-targeted drugs has been widely observed across various BC subtypes in preclinical and clinical studies.^96^ Chemoresistant BCSCs were found to inherently overexpress oncogenes such as *ALDH1A1* and efflux transporters such as *MDR1* and ABCG2. The elevated expression of the ABCG2 transporter facilitates the swift expulsion of cytotoxic drugs, thereby enhancing their resistance to chemotherapy. Additionally, MDR1 facilitates chemoresistance by modulating various cellular processes through the p53-mediated pathway. Furthermore, the increased expression of ALDH1 reduces the effectiveness of chemotherapeutic agents by converting them into less toxic molecules, consequently aiding the survival of BCSCs under treatment conditions.^97^^,^^98^

These distinctive traits have been exploited to isolate and amplify the population of BCSCs. Although most tumor cells are eradicated by chemotherapy or radiotherapy, CSCs often survive and can initiate recurrence and metastasis after treatment cessation.^99^ Lagadec et al.^100^ revealed that the prevalence of cells expressing ALDH1 increased with escalating doses of radiation. BC cells reverted to a more primitive state upon exposure to X-ray irradiation, showing an upregulation in the expression of key reprogramming factors such as Oct4, Sox2, Nanog, and Krüppel-like factor (Klf)4. Lin28 is another stem cell marker that plays a significant role in radioresistance and the maintenance of BCSCs. Its overexpression inhibits radiation-induced apoptosis by suppressing the expression of lethal (Let)-7 microRNA, which possesses tumor-suppressive functions.^101^ Lin28 promotes stemness properties through Yes-associated protein 1 (YAP1) signaling in TNBC, operating independently of Let-7 regulation.^102^ Therefore, the potential of Lin28 as a valuable biomarker beyond BCSC isolation, warrants further investigation.

Li et al.^92^ provided initial clinical evidence of the existence of chemotherapy-resistant BC-initiating cells in patients treated with conventional therapeutic drugs. This resistance was not observed in patients treated with lapatinib. Moreover, Calcagno et al.^103^ reported an increase in a cell population displaying CSC characteristics following prolonged exposure of MCF-7 cells to doxorubicin, a common chemotherapeutic agent. This stem-like population was also observed in triple-negative MDA-MB-231 cells treated with the chemotherapeutic drug vincristine.^104^ Chemoresistance toward DNA-damaging drugs (Adriamycin and cisplatin) and a poly-adenosine diphosphate (ADP)-ribose polymerase (PARP) inhibitor (olaparib) has been linked to N6-methyladenosine (m6A) modification mediated by its protein reader YTH domain containing 1 (YTHDF1).^105^ The knockdown of YTHDF1 impedes the stability of the E2F Transcription Factor (E2F)8 transcription factor by abolishing methyltransferase-like (METTL)14 activity in both MCF-7 and MDA-MB-231 cells, thereby promoting S-phase entry, DNA replication, and DNA damage repair. Furthermore, Ras-related C3 botulinum toxin substrate (RAC)1B, a small guanosine triphosphatase, is overexpressed in a subset of BC cell lines and tumor samples, where it modulates BCSC plasticity, doxorubicin resistance, and tumor-initiating capacity. Conversely, loss of RAC1B function impairs BCSC activity and enhances their chemosensitivity to doxorubicin treatment.^106^ These studies demonstrate the ability of CSCs to survive and even expand under the selective pressure of chemotherapy, consequently contributing to treatment resistance and disease recurrence.

## Therapeutic implications of breast cancer stem cells

BCs exhibit considerable heterogeneity, presenting diverse histological, molecular, and clinical features that influence disease progression and response to treatments. BC is categorized into four primary types: *in situ*, invasive, histologic subtypes, and molecular subtypes, which include luminal A, luminal B, basal-like, and HER2-enriched subtypes. Luminal subtypes of BC typically express hormone receptors, such as estrogen receptors (ERs), progesterone receptors (PRs), and/or HER2, making them more responsive to hormone-based therapies. In contrast, basal-like BCs, including TNBC, are more aggressive and do not express these hormone receptors. This leads to a lack of response to hormone therapies and a more challenging treatment landscape. These differences in receptor expression have significant implications for treatment strategies and prognosis.^107^

BCSCs are more prevalent in TNBC and HER2 subtypes compared to luminal subtypes.^12^^,^^40^ Cytotoxic chemotherapy remains the primary treatment for TNBCs to date. However, resistance to chemotherapy poses a major challenge, with many patients eventually developing resistance. This limits the long-term efficacy of current treatments. Shimo et al.^108^ demonstrated the efficacy of PARP inhibitors against TNBCs. Olaparib reduced the proportion of BCSCs with CD44^+^/CD24^−/low^/ESA^+^ cell markers, thereby suggesting its potential as an anti-CSC drug. However, Liu et al.^109^ showed that RAD51 overexpression in BRCA1-mutant and BRCA1-wild-type TNBCs can desensitize these cells to PARP inhibition. Additionally, Turdo et al.^110^ reported that dinaciclib, a cyclin-dependent kinase (CDK) inhibitor, effectively targets BCSCs in TNBC by modulating the Sam68-PARP axis and Rad51 regardless of their BRCA status.

Studies have also demonstrated the potential of epidermal growth factor receptor (EGFR) and phosphoinositide 3-kinase (PI3K) inhibitors in targeting and suppressing CSCs. For instance, eganelisib and gefitinib significantly inhibited cell proliferation, self-renewal, migration, and invasion of microRNA-induced pluripotent stem cells from T47D breast cancer cells (miPS-T47Dcm).^111^ Furthermore, combination of the EGFR/Notch bispecific antibody (prostaglandin e2 receptor (PTG)12) with the pan-PI3K inhibitor (Genentech development compound (GDC)-0941) resulted in a more potent antitumor effect in TNBC tumors. This highlights the potential of targeting these signaling pathways to enhance the response to PI3K inhibition.^112^ Targeting the androgen receptor (AR) with enzalutamide is another promising strategy, particularly because approximately half of all TNBCs express AR. Barton et al.^113^ reported that treatment with enzalutamide markedly decreased the population of BCSC-like cells in TNBC cell lines, indicating its potential to enhance the efficacy of chemotherapy.

Several drugs, such as disulfiram, mifepristone, metformin, and sulforaphane, have been repurposed as BCSC inhibitors in TNBC. For example, disulfiram, initially an anti-alcoholism medication, targeted BCSCs by reversing acquired pan-chemoresistance in TNBC cell lines.^114^ Mifepristone, originally known as a progesterone antagonist, inhibits BCSC proliferation by suppressing the expression of the stem cell transcription factor KLF5 in TNBC. This suppression is achieved through the induction of microRNA (miR)-153 expression.^115^ Metformin, typically prescribed for type 2 diabetes mellitus, also inhibits TNBC stem cells, partly through the modulation of the protein kinase (PK)A-glycogen synthase kinase (GSK)3β-KLF5 signaling pathway.^116^ Burnett et al.^117^ demonstrated that a combination of taxanes and sulforaphane could effectively eliminate stem cells in TNBC by inhibiting the translocation of the nuclear factor kappa B (NF-κB) RelA (p65) subunit and reducing NF-κB2 (p52) levels, consequently resulting in decreased transcriptional activity. The synergism of 8-quinolinol and niclosamide with paclitaxel conferred anti-CSC activity by significantly reducing cell viability and the formation of CSC-enriched mammospheres in TNBC. *In vivo* studies have also shown that co-administration of niclosamide with paclitaxel decreases tumor-circulating cells and lung cancer metastasis.^118^ Inhibitors of the sodium-potassium (Na^+^-K^+^) pump, such as cardiac glycosides traditionally used to treat heart failure, have recently shown promising effects in eliminating ancestral-like CSCs in FXYD domain containing ion transport regulator (FXYD)3-overexpressing TNBC when used alongside standard chemotherapies.^119^

Immunotherapy drugs such as pembrolizumab and atezolizumab have recently re-emerged as promising therapeutic options. Pembrolizumab is an immune-checkpoint inhibitor that targets the programmed death-1 (PD-1) receptor on the surface of T cells.^120^ Owing to its encouraging preclinical and clinical outcomes, the FDA approved the combination of this anti-PD-1 agent with chemotherapy for advanced TNBC in November 2020. Targeting co-stimulatory molecules, such as the ligand of tumor necrosis factor receptor superfamily member 4 of (OX40) alleviates immunosuppression within the tumor microenvironment by inhibiting the activity of regulatory T cells (Tregs). This process simultaneously enhances the expansion and proliferation of effector T cells, thereby strengthening the anti-tumor immune response. Moreover, OX40 agonists exhibited synergistic effects when combined with therapies such as immune checkpoint inhibitors, chemotherapy, and radiotherapy. These combinations could amplify immune responses, leading to significant improvements in survival rates for patients with BC.^120^^,^^121^ As reviewed by Tabana et al.,^120^ the progress in immunotherapy for BC goes beyond the PD-1/programmed death ligand 1 (PD-L1) axis, novel targets such as stimulator of interferon genes (STING), toll-like receptors (TLRs), and colony-stimulating factors/receptor (CSF-1/CSF-1R) pathways, which hold promise for enhancing immunotherapy outcomes. Therefore, further investigation into these therapeutic modalities should be largely explored.

Unlike invasive subtypes, luminal BC subtypes typically have lower CSC content and are predominantly enriched with CD44^−/low^CD24^+^ populations. These subtypes are typically responsive to hormone therapy. However, ER^+^ tumors can develop endocrine resistance and display elevated CSC populations. Multiple mechanisms underlying endocrine resistance have also been identified, including somatic alterations, epigenetic modifications, metabolic reprogramming, and changes in the tumor microenvironment. Endocrine therapies, such as selective ER modulators (SERMs) (e.g., tamoxifen), selective ER downregulators (SERDs) (e.g., fulvestrant), and aromatase inhibitors (AIs) (e.g., anastrozole), are commonly used. However, their efficacy is often compromised as resistance develops.^122^

Inhibitors targeting CDK4/6, PI3K, and mammalian target of rapamycin (mTOR) signaling have been employed to address relapses in patients undergoing endocrine therapy. However, their efficacy is limited by toxicity and resistance. Moreover, the combination of endocrine therapy with inhibitors targeting pathways such as Notch, hypoxia-inducible factor (HIF), and integrin/AKT – which maintain CSCs – effectively reduces the self-renewal capacity of hormone receptor-positive BC cells. Thus, it may offer a potentially effective therapeutic strategy.^122^^,^^123^ Endocrine-resistant BCs and CSCs have demonstrated susceptibility to tumor necrosis factor-related apoptosis-inducing ligand (TRAIL) agonists through the degradation of an apoptosis inhibitor (cellular FADD-like interleukin-1β converting enzyme [FLICE]-like inhibitory protein [cFLIP]), which is mediated by the c-Jun N-terminal kinase (JNK) pathway.^124^ Ozaki et al.^125^ recently showed that a luminal B subtype with high p62 and Aldehyde dehydrogenase 1 familymember a3 (ALDH1A3) expression exhibited reduced sensitivity to radiotherapy. However, the combination of p62 inhibition with radiation therapy significantly decreased the formation of tumorspheres in ALDH1^+^ luminal B. Thus, this combination strategy presents a more promising avenue for improving treatment outcomes.

HER2 is highly expressed in the ALDH^+^ BCSC population and contributes to the tumorigenesis and metastasis of luminal BC. HER2 expression is regulated by the Notch signaling pathway and promotes the proliferation of tumor-initiating cells.^40^^,^^126^ HER2 inhibitors commonly used in therapy, such as trastuzumab, pertuzumab, lapatinib, and trastuzumab emtansine (T-DM1), have shown promising outcomes. Nonetheless, certain HER2-positive BCs may develop resistance to these drugs.^127^ Various strategies for counteracting this chemoresistance have been comprehensively discussed by Qiu et al.^128^ Trastuzumab, a monoclonal antibody against the HER2 receptor, effectively targeted both non-BCSCs and ALDH^+^ BCSCs in HER2-positive breast carcinoma cells. Diessner et al.^129^ showed that an antibody-drug conjugated cytotoxic agent targeted CSCs by increasing autophagy and facilitating internalization of HER2 from the cell surface in HER2-positive BC cells. Resistance mechanisms may also stem from the loss of the phosphatase and tensin homolog (PTEN) or PI3K activation, resulting in EMT or subtype switching.^130^^,^^131^ Furthermore, co-administration of pan-PI3K inhibitors such as XL147, LY-294002, and NVP-BKM120 could prevent trastuzumab resistance. These inhibitors acted synergistically with trastuzumab to eliminate BCSCs by suppressing PI3K activation.^128^

## Challenges and prospects of breast cancer stem cells

BCSCs play a pivotal role in cancer progression and recurrence, owing to their unique properties, including self-renewal, quiescence, tumor initiation, and metastasis promotion. These traits make them central to tumor growth and enable them to evade conventional therapies, which typically target rapidly proliferating cells. Therefore, isolating and studying BCSCs can provide invaluable insights into the mechanisms underlying therapeutic resistance and facilitate the identification of potential therapeutic targets. However, the heterogeneity of BCSCs across different BC subtypes and the absence of specific biomarkers for distinguishing these subtypes hinders the development of more effective treatments.

Although immunotherapy has shown promise in treating invasive BC, surviving cells within the tumor microenvironment undergo metabolic shifts and activate immune escape mechanisms, consequently evading treatment. Furthermore, current treatments primarily rely on pathological and hormone receptor profiles, which often fail to address the mechanisms underlying resistance and relapse. This consequently limits their long-term efficacy. Despite therapeutic advancements, cancer cell adaptability and subsequent therapy-induced resistance remain substantial barriers to consistent and effective treatment outcomes. In addition, the translation of preclinical findings to clinical settings poses significant challenges that require rigorous validation to ensure patient safety and efficacy. Emerging evidence has also suggested that therapeutic resistance is closely linked to adverse immune response and metabolic reprogramming. Therefore, understanding the complex regulatory networks involved in genetic, metabolic, and immune adaptations within the tumor microenvironment is critical for unraveling how they contribute to chemoresistance and stemness. Gaining these comprehensive and integrated insights is vital for advancing the development of innovative and more effective therapeutic strategies tailored to patients with BC.

## Authors contribution

Peik Lin Teoh: conceptualization, methodology, investigation, writing – review & editing; Nurshafiqah Saini: investigation, writing – original draft. All the authors critically revised and approved the final version of the manuscript. All the authors critically revised and approved the final version of the manuscript.

## Ethics statement

None.

## Data availability statement

The datasets used in the current study are available from the corresponding author on reasonable request.

## Declaration of Generative AI and AI-assisted technologies in the writing process

During the preparation of this work, the author(s) used Chat Generative Pre-trained Transformer (ChatGPT) in order to improve clarity, coherence, and readability of the text. After using this tool, the authors reviewed and edited the content as needed and take full responsibility for the content of the publication.

## Funding

This work was supported by the Universiti Malaysia Sabah (No.: SDK0440-2018).

## Conflict of interest

The authors declare that they have no known competing financial interests or personal relationships that could have appeared to influence the work reported in this paper.
